# Peptides derived from Kex2-processed repeat proteins are widely distributed and highly diverse in the Fungi kingdom

**DOI:** 10.1186/s40694-020-00100-5

**Published:** 2020-07-01

**Authors:** Maiko Umemura

**Affiliations:** Bioproduction Research Institute, National Institute of Advanced Industrial Science and Technology (AIST), Ibaraki, 305-8566 Japan

**Keywords:** Ribosomally synthesized and post-translationally modified peptides (RiPPs), Kex2-processed repeat proteins (KEPs), Tandem-repeat sequences, Secretory peptides, Pheromones, Hormones

## Abstract

**Background:**

Recently, a gene cluster responsible for biosynthesis of ustiloxin in *Aspergillus flavus* was identified as the first case of a ribosomally synthesized and post-translationally modified peptide (RiPP) synthetic pathway in Ascomycota. RiPPs are biosynthesized from precursor peptides, which are processed to produce the RiPP backbone (core peptides) for further modifications such as methylation and cyclization. Ustiloxin precursor peptide has two distinctive features: a signal peptide for translocation into the endoplasmic reticulum and highly repeated core sequences cleaved by Kex2 protease in the Golgi apparatus. On the basis of these characteristics, the ustiloxin-type RiPP precursor peptides or Kex2-processed repeat proteins (KEPs) in strains belonging to the Fungi kingdom were computationally surveyed, in order to investigate the distribution and putative functions of KEPs in fungal ecology.

**Results:**

In total, 7878 KEPs were detected in 1345 of 1461 strains belonging to 8 phyla. The average number of KEPs per strain was 5.25 in Ascomycota and 5.30 in Basidiomycota, but only 1.35 in the class Saccharomycetes (Ascomycota) and 1.00 in the class Tremellomycetes (Basidiomycota). The KEPs were classified into 838 types and 2560 stand-alone ones, which had no homologs. Nearly 200 types were distributed in more than one genus, and 14 types in more than one phylum. These types included yeast α-mating factors and fungal pheromones. Genes for 22% KEPs were accompanied by genes for DUF3328-domain-containing proteins, which are indispensable for cyclization of the core peptides. DUF3328-domain-containing protein genes were located at an average distance of 3.09 genes from KEP genes. Genes for almost all (with three exceptions) KEPs annotated as yeast α-mating factors or fungal pheromones were not accompanied by DUF3328-domain-containing protein genes.

**Conclusion:**

KEPs are widely distributed in the Fungi kingdom, but their repeated sequences are highly diverse. From these results and some examples, a hypothesis was raised that KEPs initially evolved as unmodified linear peptides (*e.g.*, mating factors), and then those that adopted a modified cyclic form emerged (*e.g.*, toxins) to utilize their strong bioactivity against predators and competitive microorganisms.

## Background

**Fig. 1:**
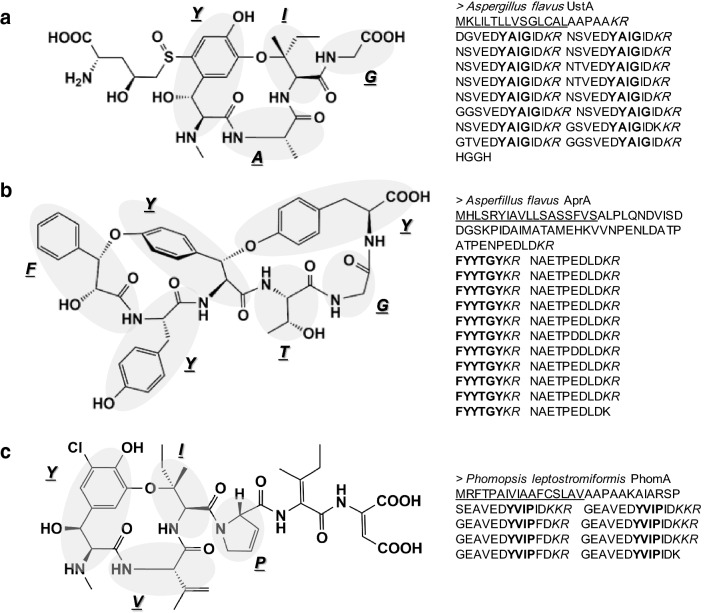
Structures and precursor peptide sequences of ustiloxin-type ribosomally synthesized and post-translationally modified peptides (RiPPs). **a** Ustiloxin B from *Aspergillus flavus*, **b** asperipin-2a from *A. flavus*, and **c** phomopsin A from *Phomopsis leptostromiformis*. The core peptides, which become the RiPP backbone, are shown in bold; recognition sites for Kex2 protease in the precursor peptides are italicized. The signal peptides for translocation into the endoplasmic reticulum are underlined

Repertoires of secretory proteins and peptides have been characterized in fungi and nematodes as signaling molecules and effectors to manipulate the immune systems of host plants [1–6]. We identified an *Aspergillus flavus* gene cluster responsible for biosynthesis of ustiloxin B [7], which is a cyclic peptide of five amino acids (Fig. 1a) [8]. This was the first case of a synthetic pathway for a ribosomally synthesized and post-translationally modified peptide (RiPP) in ascomycetes [7], with the genes for amatoxin biosynthesis as the first RiPP synthetic pathway in fungi [9]. RiPPs are a class of secondary metabolites produced by a wide range of organisms including bacteria [10], archaea [11] and fungi [9]. They are biosynthesized from precursor peptides, which contain sequences used to produce the RiPP backbone structures (core peptides), with various posttranslational modifications such as cyclization, sulfation, conversion of N-terminal glutamate to pyroglutamate and C-terminal amidation [12]. The precursor peptide of ustiloxin has two distinctive features: (1) a signal peptide for the translocation into the endoplasmic reticulum (ER) and (2) highly repeated core peptide sequences processed by Kex2 protease localized in the Golgi apparatus. The genes for two additional proteins homologous to each other, whose functions are unknown but indispensable for cyclization of the core peptides, were found in the vicinity of the gene encoding the ustiloxin precursor peptide [7, 13]. These two cyclization factors have a domain called DUF3328. Based on these three characteristics, we revealed that the ustiloxin-like biosynthetic pathways are widely distributed in the genus *Aspergillus*, although the core peptide sequences are diversified into more than 40 distinct types [13]. From information on a computationally detected pathway, we identified a novel cyclic peptide, asperipin-2a (Fig. 1b), from *A. flavus* [13]. We named this class of RiPP biosynthetic pathways “ust-RiPS” (ustiloxin-type RiPP synthesis).

Ustiloxin B and asperipin-2a are not the only fungal RiPPs synthesized by the ust-RiPS pathways [14]. Johnson et al. revealed that the fungal endophytes of grasses belonging to the genus *Epichloë* secrete a variety of cyclic peptides, which are produced from a ustiloxin-type precursor peptide, GigA [15]. Ding et al. identified the RiPP biosynthetic pathway for phomopsins (Fig. 1c) in *Phomopsis leptostromiformis*, whose precursor peptide shares the two features of ustiloxin and asperipin-2a precursor peptides and its gene is accompanied by five DUF3328-domain-containing protein genes [16]. The phomopsin core peptide is YVIP, in which amino acids at the first and third positions are the same as in the core peptides of ustiloxins A and B (YVIG and YAIG, respectively) [17]; all these peptides are cyclized between the side chains of Tyr and Ile (Fig. 1). From the structure of the three ust-RiPS compounds, DUF3328-domain-containing proteins are considered to catalyze the cyclization through ether bond formation directly in the aromatic ring of Y [16, 18, 19]. A tyrosinase is also indispensable for the cyclization of the core peptides of ustiloxins and phomopsins [16, 18]. Ding et al. further detected 27 phomopsin-like precursor peptides whose genes are accompanied by DUF3328-domain-containing protein genes in the Dikarya subkingdom [16]. They called this class of putative compounds “dikaritins” [16].

The above data strongly support the idea that ustiloxin-type precursor peptides and the corresponding biosynthetic pathways are widely distributed among fungi and play important roles in their ecology. The ectomycorrhizal fungus *Laccaria bicolor* produces MiSSP8 from the repetitive precursor peptide cleaved by Kex2 recognition sites, and the knockdown mutant of MiSSP8-encoding gene is strongly impaired in their mycorrhization ability with *Populus* [20]. The maize pathogenic fungus *Ustilago maydis* secretes at least 8 similar proteins from a repetitive protein Rep1 cleaved by Kex2, which are water-repellents and necessary to form aerial hyphae [21]. The *U. maydis* double knockout of *rep1* and *hum3*, both encode repetitive proteins with Kex2 sites, severely affects the fungus pathogenicity against maize [22]. Another effector Rsp3 from *U. maydis*, which blocks the antifungal activity of mannose-binding maize proteins, comprises a moiety of repeated sequences cleaved by Kex2 [23]. Candidalysin or Ece1p from the human pathogenic fungus *Candida albicans* directly damages epithelial membrane and enhance membrane permeabilization [24]. Ece1p itself is not repeated but one of the eight peptide moieties cleaved by Kex2 from its precursor protein, Ece1, in which other three moieties are repeated. Marquer et al. recently computationally surveyed 250 fungal genomes and detected 1183 proteins sharing the two features of ustiloxin-type precursor peptides, which they called KEPs (Kex2-processed repeat proteins) [25]. The detected KEPs contained a group of Ascomycota α-type sexual pheromones containing the motif of repeated XA or XP dipeptides recognized by STE13 protease after the Kex2 and Kex1 recognition sites (KR, RR or KK) [26–29], and KEPs having the same motifs were also found in Basidiomycota and Glomeromycotina [25]. Their abbreviation “KEP” is hereafter adopted for the secretory proteins having repetitive sequences by Kex2 protease, including ustiloxin-type RiPP precursor peptides.

Compared to these examples of linear peptides from KEPs, the specific functions or roles of cyclic peptides modified from KEPs in fungal ecology have not been well-characterized. Johnson et al. deleted the gene encoding GigA, which resulted in elimination of all GigA-origin cyclic peptides (epichloëcyclins), but they did not observe any phenotypic impacts of the deletion on the host grass [15]. The deletion of the ustiloxin biosynthetic cluster (composed of 15 co-regulated genes) or of the asperipin-2a precursor peptide does not result in any phenotypic changes in comparison with the wild type in *A. flavus* [7, 13]. Ustiloxins and phomopsins strongly inhibit microtubule assembly [30–32]; however, the amount of ustiloxins produced by *Ustilaginoidea virens*, from which the compounds were originally identified [8, 33], does not correlate with pathogenicity of the strain against rice [34].

Here, to grasp the overall picture regarding KEPs more comprehensively and in relation to the cyclization factor DUF3328-domain-containing proteins, 1461 strains belonging to 8 phyla in the Fungi kingdom were mined and detected KEPs were characterized on the basis of their repeated sequences, functional annotations, and the existence of genes for DUF3328-domain-containing proteins in the vicinity of KEP-encoding genes. The result illustrates that KEPs are widely distributed in the Fungi kingdom, but the core peptide sequences are highly diverse. From the observations and published reports, the evolution and roles of KEPs in fungal ecology are discussed.

## Results

### KEPs are widely distributed in the Fungi kingdom

**Fig. 2:**
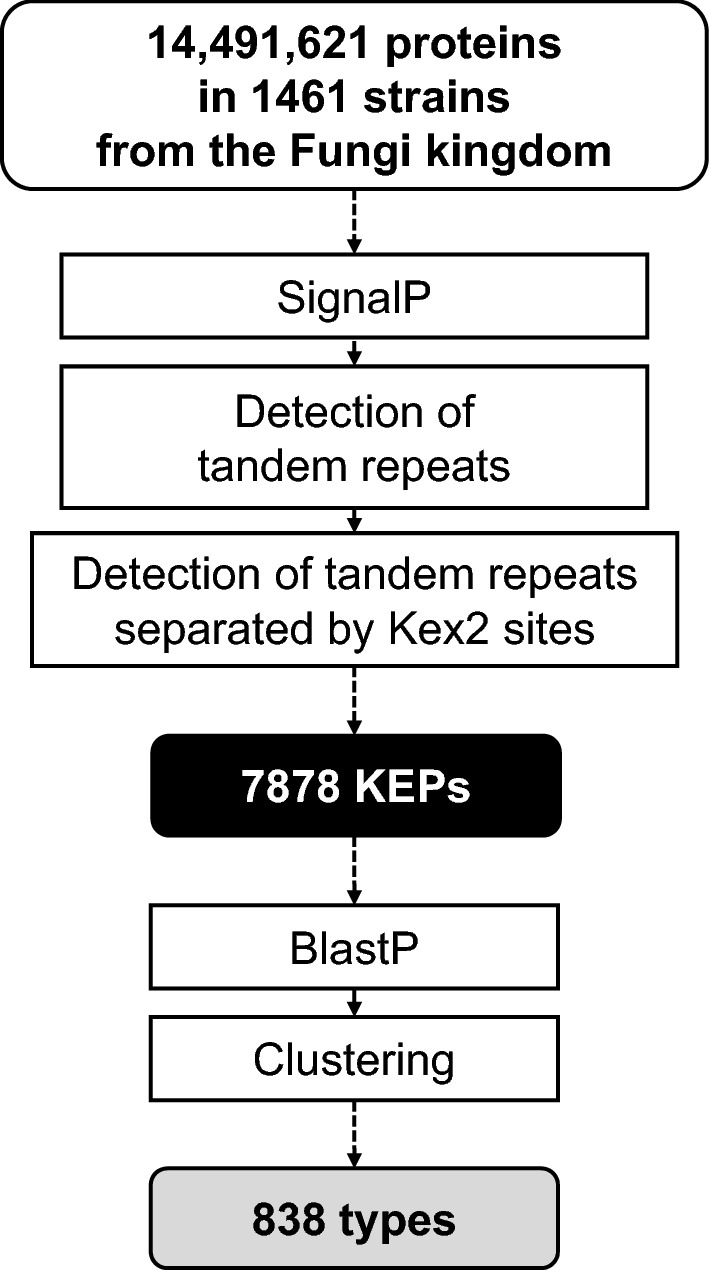
The procedure of a computational survey of Kex2-processed repeat proteins (KEPs). Data are shown in rounded rectangles and processes in rectangles with corners

KEPs were mined against 1461 whole genome assemblies of the strains belonging to 8 phyla in the Fungi kingdom (Table 1). The mining was based on two characteristics of ustiloxin-type precursor peptides, *i.e.*, (1) an ER signal peptide and (2) highly repeated sequences separated by Kex2 recognition sites (Fig. 2). Ascomycota (filamentous fungi and budding yeasts) is the largest phylum, containing 1024 strains, and Basidiomycota (mushrooms) is the second largest one, with 317 strains. The Microsporidia phylum contains 39 strains, which are obligate intracellular parasites of vertebrates and invertebrates. Mucoromycota also contain 39 strains, mainly from the Glomeromycotina and Mucoromycotina subphyla (Additional file 1: Table S1). The Chytridiomycota, Zoopagomycota, Cryptomycota, and Blastocladiomycota contain small numbers of strains (< 20). Two assemblies are classified just as Fungi (GCA_000836255.1 and GCA_002003505.1; “Not classified” in Table 1). Among the surveyed 1461 strains, 171 were also surveyed by Marquer et al. [25], whilst 1290 strains were newly surveyed in this study (Table 1).

**Table 1:** Phyla from the Fungi kingdom surveyed in this study

Phylum	Strains			KEPs		
	Surveyed^a^	Found	Genera	Total^b^	Average	SD
Ascomycota	1024 (109)	970	216	5372 (1647)	5.25	4.78
Basidiomycota	317 (44)	271	122	1680 (103)	5.30	6.72
Mucoromycota	39 (7)	38	18	357 (0)	9.15	5.81
Microsporidia	39 (7)	29	18	68 (0)	1.74	2.34
Chytridiomycota	19 (1)	14	12	178 (0)	9.37	10.64
Zoopagomycota	16 (2)	16	11	185 (0)	11.56	10.58
Cryptomycota	3 (1)	3	2	14 (0)	4.67	1.53
Blastocladiomycota	2 (0)	2	2	12 (0)	6.00	1.41
Not classified	2 (0)	2	–	12 (8)	6.00	4.24
Total	1461	1345	401	7878	5.39	5.55

Latest assemblies registered in NCBI on October 9, 2019 were used

^a^The number of strains surveyed by Marquer et al. [25] (in parentheses)

^b^The number of KEP-encoding genes accompanied by DUF3328-domain containing protein genes (in parentheses)

**Fig. 3:**
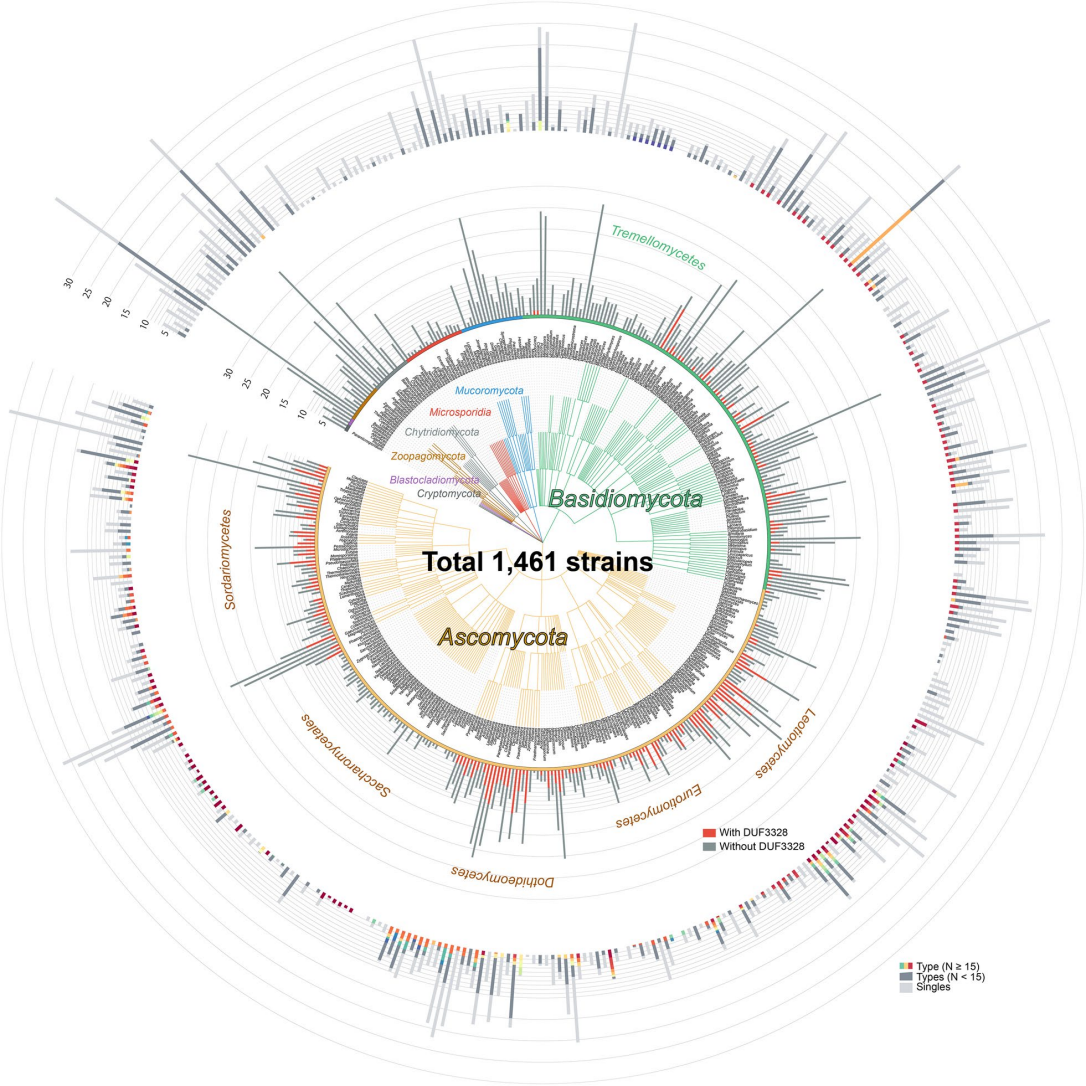
Distribution of Kex2-processed repeat proteins (KEPs) in the Fungi kingdom shown on a taxonomic tree of the surveyed strains. The phyla are shown by colored edges and circle segments outside the tree. Outer bars show the average number of KEPs per genus, colored by types containing at least 15 KEPs (gradation from red to yellow to green to blue); types containing fewer than 15 KEPs are shown in dark grey and stand-alone KEPs without any homologs in light grey. Inner bars also show the average numbers of KEPs per genus, but are colored by the KEPs with (red) and without (dark grey) DUF3328-domain-containing proteins encoded within the 15 genes closest to the KEP-encoding gene. The figure in a vector format is available (Additional file 2)

Among 14,491,621 proteins annotated in the 1461 assemblies, 7.7% (1,123,012) had ER signal peptides, and 6.2% (69,264) among them had any tandem-repeat sequences. Finally, a total of 7878 KEPs were detected from 1345 out of the 1461 strains (Table 1). These KEPs were widely distributed in the Fungi kingdom (Fig. 3). The average number of KEPs per strain was 5.39, which was close to those in Ascomycota (5.25) and Basidiomycota (5.30), although standard deviation was higher in the latter (6.72) than in the former (4.78). The Zoopagomycota phylum was the most densely populated by KEPs (11.56 per strain), followed by Chytridiomycota (9.37) and Mucoromycota (9.15). The large population in Zoopagomycota and Chytridiomycota came from some strains with abundant KEPs, as reflected in the high standard deviations (> 10). The most populated strain was *Basidiobolus meristosporus* CBS 931.73 (Zoopagomycota), which possessed 43 KEPs, although more than half of them were duplicated or belonged to the same types, as described later. In contrast to these two phyla, strains in Mucoromycota were evenly populated by KEPs (standard deviation 5.81, close to 5.55 among all strains). On the other hand, strains in Microsporidia were conspicuously devoid of KEPs (1.74 per strain).

KEPs were not detected in 116 strains from Ascomycota (54 strains), Basidiomycota (46), Microsporidia (10), Chytridiomycota (5), and Mucoromycota (1). It is noteworthy that among the 54 Ascomycota strains 24 belonged to the class Saccharomycetes, and among the 46 Basidiomycota strains 46 belonged to the class Tremellomycetes. The average number of KEPs per strain was 1.35 in Saccharomycetes and 1.00 in Tremellomycetes; both values were lower than that of Microsporidia (Additional file 1: Table S1). The scarcity of KEPs in these two classes and in the Microsporidia can be seen in Fig. 3. This result is in accordance with the previous report that yeast-like or unicellular fungi displayed a smaller number of KEPs than filamentous fungi [25].

The detection procedure of KEPs was fundamentally the same as in the previous study [25], except that proteins were excluded when they contained separated sequence(s) longer than 100 a.a. or all repeated sequences were shorter than 8 a.a. in this study, because here KEPs were surveyed based on the characteristics of the ustiloxin precursor peptides. Mainly due to this difference, 536 KEPs (45%) of 1183 reported in the previous study were not detected in this study. On the other hand, in the overlapped 171 strains, 684 KEPs (53%) of 1281 detected in this study were not reported in the previous study (Additional file 1: Table S1); the reason for this is unknown because no notable aspect was observed in the KEP sequences only detected in this study.

### KEPs are highly diverse in the Fungi kingdom

Of the 7878 KEPs, 5318 were classified into 838 types according to e-values and coverages evaluated by a BlastP search against a database containing all 7878 KEPs. The remaining 2560 KEPs were assigned the stand-alone status, because they did not have any homologs. The largest type comprised 191 KEPs and 68 types with 15 KEPs or more, whereas 769 out of the 838 types comprised 14 KEPs or fewer and 376 out of the 769 types were composed of just 2 KEPs (Additional file 3: Table S2).

**Fig. 4:**
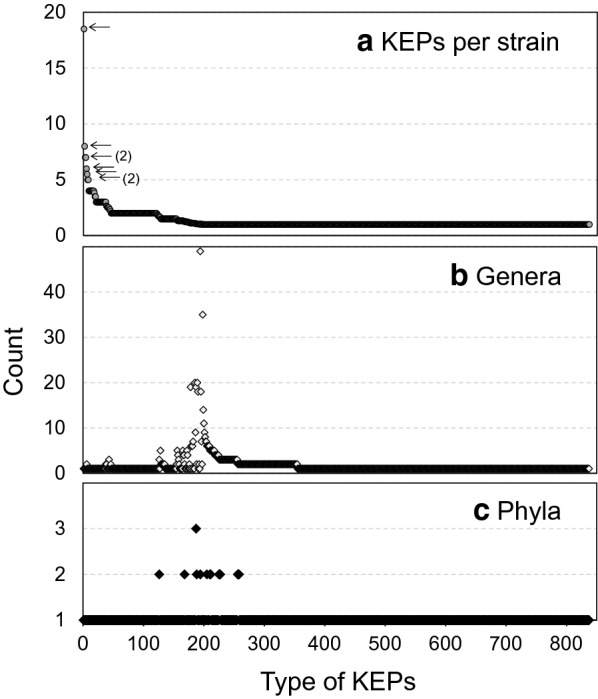
Counts of Kex2-processed repeat proteins (KEPs) according to type, ordered by the number of KEPs per strain. **a** Number of KEPs per strain. The top 8 types with five or more KEPs per strain are indicated by small arrows. The numbers in parentheses next to the arrows indicate the numbers of types at the positions when they are not 1. **b** Number of genera possessing KEPs, and **c** number of phyla possessing KEPs. The stand-alone KEPs are not shown in this figure

**Fig. 5:**
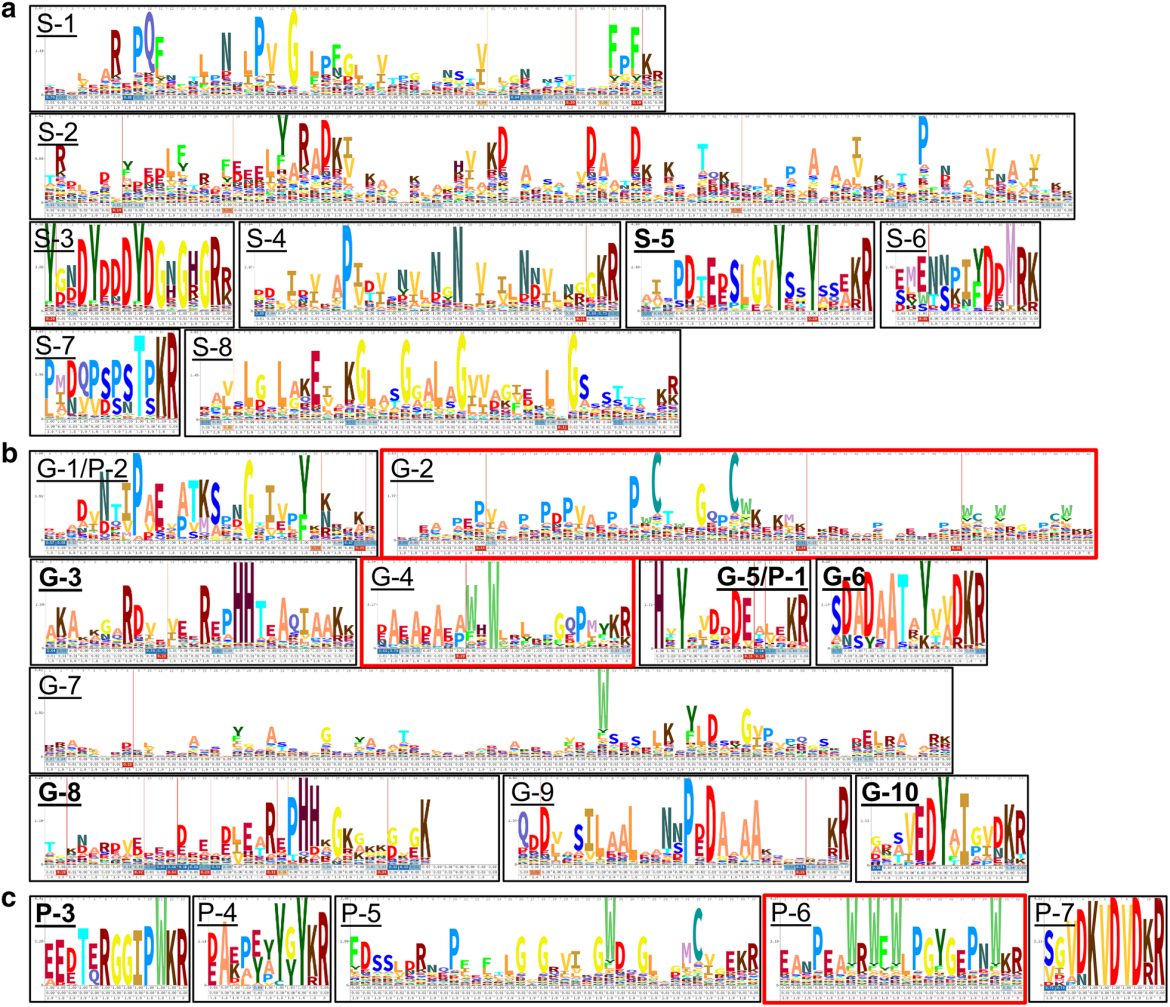
Hidden Markov model (HMM) profiles of major Kex2-processed repeat proteins (KEPs) corresponding to Fig. 4 and Table 2. **a** Top 8 types by number of KEPs per strain, **b** top 10 types by number of genera possessing KEPs, and **c** top 7 types by number of phyla possessing KEPs. The top two types in **c** are shown in **b** as G-5/P-1 and G-1/P-2, respectively. Type G-4 annotated as yeast α-mating factors and types G-2 and P-6 annotated as fungal pheromones are framed in bold red. The labels are in bold when DUF3328-domain-containing proteins are encoded in the vicinity of KEP-encoding genes. Type G-10 includes the ustiloxin precursor peptide

However, these numbers are strongly affected by the biased distribution of strains with assembled genomes among genera. For example, the genomes of 120 strains are registered for *Saccharomyces cerevisiae*, but only one for *Zancudomyces culisetae*. To alleviate this bias, we ordered the 838 KEP-types by the number of the KEPs of each type per strain (Fig. 4). The top 8 KEP-types comprised 5 or more KEPs per strain, among which the most abundant type had 18.5 KEPs per strain, probably owing to gene duplications (Fig. 4a). For example, the strain *Peniophora* sp. CBMAI 1063 has 20 KEPs of type S-1, and three of which (VDB83965.1, VDB83975.1, VDB83983.1) are almost identical to each other with e-values of 0 with more than 86% coverage by BlastP searches (Additional file 4: Table S3). These duplications occurred in one or two strains from the same genus, typically from the Basidiomycota, Chytridiomycota, and Zoopagomycota, except type S-6 (#91) where the KEPs were found in two genera, *Neocallimastix* and *Piromyces* (Table 2). The most populated strain, *B. meristosporus* CBS 931.73, had 7 KEPs of type S-3 and 18 KEPs whose genes were present in 2 or 3 copies. These KEPs may have functions specific to particular strains. The repeated sequences in the KEPs of these types are shown in a profile of a hidden Markov model (HMM) in Fig. 5a. No sequence similarities were found among the 8 types. The most duplicated type (S-1) was annotated as having the Pfam motifs YukD (the WXG100 protein secretion system), NifZ (nitrogen fixation operon) and DUF5109 (the domain for binding or recognition of ligands at the C-terminus of a putative glycosyl-hydrolase family) by an HMM search (Table 2).

**Table 2:** Characterization of KEP types

Label^a^	Type ID^b^	Count											
		FRiPS factors	Strains	KEPs per strain	Genera	Phyla	Yeast α-mating factors^c^	Fungal pheromones^d^	With DUF3328^e^	Without DUF3328^f^	Genera	Phyla	HMM profiles
Top 8 types sorted by number of KEPs per strain													
S-1	#22	37	2	18.50	1	1	0	0	0	37	Peniophora	Basidiomycota	DUF5109, NifZ, YukD
S-2	#139	8	1	8.00	1	1	0	0	0	8	Hypsizygus	Basidiomycota	SseB_C, FACT-Spt16_Nlob, DUF445
S-3	#158	7	1	7.00	1	1	0	0	0	7	Basidiobolus	Zoopagomycota	
S-4	#171	7	1	7.00	1	1	0	0	0	7	Fibularhizoctonia	Basidiomycota	TOM13, ribosomal_L24
S-5	#80	12	2	6.00	1	1	0	0	8	4	Sistotremastrum	Basidiomycota	DUF4170, Eryth_link_C, DUF3756
S-6	#91	11	2	5.50	2	1	0	0	0	11	Neocallimastix, Piromyces	Chytridiomycota	Chitin_bind_1
S-7	#107	10	2	5.00	1	1	0	0	0	10	Batrachochytrium	Chytridiomycota	
S-8	#203	5	1	5.00	1	1	0	0	1	4	Rickenella	Basidiomycota	
Top 10 types sorted by number of genera having KEPs													
G-1/P-2	#7	64	62	1.03	49	2	0	0	0	64	Termitomyces, Moniliophthora, Auriculariopsis, Crucibulum, Trametes, Sphaerobolus, Laccaria, Hypsizygus, Sanghuangporus, Hypholoma, Gloeophyllum, Pleurotus, Dichomitus, Armillaria, Laetiporus, Lentinus, Coprinopsis, Heliocybe, Dendrothele, Panaeolus, Phlebiopsis, Peniophora, Schizopora, Hebeloma, Pterula, Galerina, Schizophyllum, Ganoderma, Antrodiella, Pyrrhoderma, Bondarzewia, Rickenella, Obba, Postia, Pluteus, Neolentinus, Polyporus, Plicaturopsis, Punctularia, Heterobasidion, Dentipellis, Gymnopilus, Stereum, Fibularhizoctonia, Fibroporia, Cyphellophora, Grifola, Fomitiporia, Gelatoporia	Basidiomycota, Ascomycota	E_Pc_C, Pab87_oct, Osteoregulin, ATXN-1_C, ChlamPMP_M, Cytomega_UL20A, DUF4452, DUF5542, TubC_N, TruB_N, TruB_C, MATH, DUF2188, Motilin_assoc, Sid-5, Str_synth, B_lectin, Phage-tail_3, DUF1648, DUF421, UPF0258, CSD2, SspK, DNA_gyraseA_C, FtsK_alpha, TAFH, gp37_C
G-2	#2	138	137	1.01	35	1	0	121	1	137	Diplocarpon, Botryotinia, Fusarium, Phialocephala, Pseudogymnoascus, Verticillium, Botrytis, Metarhizium, Coleophoma, Purpureocillium, Lachnellula, Marssonina, Coniella, Trichoderma, Neurospora, Rutstroemia, Sporothrix, Monilinia, Ophiostoma, Pezoloma, Sclerotinia, Sphaerosporella, Colletotrichum, Amorphotheca, Coniochaeta, Thermothielavioides, Podospora, Madurella, Escovopsis, Hyaloscypha, Scytalidium, Chlorociboria, Venustampulla, Cadophora, Sodiomyces	Ascomycota	Trefoil, DUF2390, Trypan_PARP, PknG_rubred
G-3	#6	64	59	1.08	20	1	0	0	57	7	Botryotinia, Phialocephala, Botrytis, Lachnellula, Trichoderma, Rutstroemia, Pezoloma, Hyaloscypha, Scytalidium, Cadophora, Penicillium, Rhynchosporium, Talaromyces, Fonsecaea, Verruconis, Capronia, Cladophialophora, Phialophora, Exophiala, Oidiodendron	Ascomycota	
G-4	#1	191	181	1.06	20	1	174	0	0	191	Saccharomyces, Cyberlindnera, Lachancea, Nakaseomyces, Kazachstania, Kluyveromyces, Naumovozyma, Wickerhamomyces, Zygosaccharomyces, Clavispora, Tetrapisispora, Eremothecium, Debaryomyces, Hanseniaspora, Vanderwaltozyma, Millerozyma, Torulaspora, Saccharomycodes, Metschnikowia, Suhomyces	Ascomycota	CLP1_P, Ribos_L4_asso_C, Cytochrome_P460, MF_alpha_N, MF_alpha, Phage_1_1
G-5/P-1	#24	35	33	1.06	19	3	0	0	16	19	Botryotinia, Botrytis, Coleophoma, Rutstroemia, Sclerotinia, Colletotrichum, Aspergillus, Penicillium, Dendrothele, Bondarzewia, Pluteus, Heterobasidion, Phialemoniopsis, Cordyceps, Beauveria, Parastagonospora, Leptosphaeria, Psilocybe, Blyttiomyces	Ascomycota, Basidiomycota, Chytridiomycota	CW_binding_1, DUF1871, Fe_hyd_SSU, HTH_19, ESSS, Fibrinogen_BP, DUF3976, Nuc_N, RHS_repeat, ATP1G1_PLM_MAT8, SHS2_FTSA
G-6	#13	49	43	1.14	19	1	0	0	46	3	Pseudogymnoascus, Rutstroemia, Colletotrichum, Aspergillus, Penicillium, Rhynchosporium, Talaromyces, Chaetomium, Cordyceps, Blastomyces, Paracoccidioides, Monosporascus, Hypoxylon, Histoplasma, Emergomyces, Beauveria, Torrubiella, Emmonsia, Ophiocordyceps	Ascomycota	ID, Fibrinogen_BP, CRA_rpt, CAP160, FliC, DUF4485, Novirhabdo_Nv
G-7	#12	50	48	1.04	18	1	0	0	1	49	Verticillium, Neurospora, Colletotrichum, Thermothielavioides, Sodiomyces, Aspergillus, Capronia, Exophiala, Phaeoacremonium, Valsa, Cytospora, Phialemoniopsis, Gaeumannomyces, Magnaporthiopsis, Thermothelomyces, Umbilicaria, Chaetomium, Eurotiomycetes	Ascomycota	SAP, Hydrophobin_2, Ish1, HeH, Slx4
G-8	#15	49	48	1.02	18	1	0	0	45	4	Pseudogymnoascus, Sporothrix, Colletotrichum, Coniochaeta, Thermothielavioides, Phialemoniopsis, Pyrenophora, Pyricularia, Grosmannia, Stagonospora, Parastagonospora, Paraphaeosphaeria, Bipolaris, Exserohilum, Epicoccum, Ascochyta, Pyrenochaeta, Periconia	Ascomycota	PAP_assoc, WHH
G-9	#16	48	48	1.00	14	1	0	0	0	48	Phialocephala, Verruconis, Pyrenophora, Parastagonospora, Bipolaris, Exserohilum, Epicoccum, Ascochyta, Pyrenochaeta, Pestalotiopsis, Alternaria, Stemphylium, Corynespora, Venturia	Ascomycota	CARD_2, T2SSM_b, DUF3953
G-10	#19	45	45	1.00	11	1	0	0	41	4	Metarhizium, Neurospora, Aspergillus, Penicillium, Zymoseptoria, Cordyceps, Beauveria, Ophiocordyceps, Sordaria, Ustilaginoidea, Pseudocercospora	Ascomycota	DUF2020, VIT, DUF3983
Top 7 types sorted by number of phyla having KEPs^g^													
G-5/P-1	#24	35	33	1.06	19	3	0	0	16	19	Botryotinia, Botrytis, Coleophoma, Rutstroemia, Sclerotinia, Colletotrichum, Aspergillus, Penicillium, Dendrothele, Bondarzewia, Pluteus, Heterobasidion, Phialemoniopsis, Cordyceps, Beauveria, Parastagonospora, Leptosphaeria, Psilocybe, Blyttiomyces	Ascomycota, Basidiomycota, Chytridiomycota	CW_binding_1, DUF1871, Fe_hyd_SSU, HTH_19, ESSS, Fibrinogen_BP, DUF3976, Nuc_N, RHS_repeat, ATP1G1_PLM_MAT8, SHS2_FTSA
G-1/P-2	#7	64	62	1.03	49	2	0	0	0	64	Termitomyces, Moniliophthora, Auriculariopsis, Crucibulum, Trametes, Sphaerobolus, Laccaria, Hypsizygus, Sanghuangporus, Hypholoma, Gloeophyllum, Pleurotus, Dichomitus, Armillaria, Laetiporus, Lentinus, Coprinopsis, Heliocybe, Dendrothele, Panaeolus, Phlebiopsis, Peniophora, Schizopora, Hebeloma, Pterula, Galerina, Schizophyllum, Ganoderma, Antrodiella, Pyrrhoderma, Bondarzewia, Rickenella, Obba, Postia, Pluteus, Neolentinus, Polyporus, Plicaturopsis, Punctularia, Heterobasidion, Dentipellis, Gymnopilus, Stereum, Fibularhizoctonia, Fibroporia, Cyphellophora, Grifola, Fomitiporia, Gelatoporia	Basidiomycota, Ascomycota	E_Pc_C, Pab87_oct, Osteoregulin, ATXN-1_C, ChlamPMP_M, Cytomega_UL20A, DUF4452, DUF5542, TubC_N, TruB_N, TruB_C, MATH, DUF2188, Motilin_assoc, Sid-5, Str_synth, B_lectin, Phage-tail_3, DUF1648, DUF421, UPF0258, CSD2, SspK, DNA_gyraseA_C, FtsK_alpha, TAFH, gp37_C
P-3	#165	7	7	1.00	5	2	0	0	4	3	Fonsecaea, Cladophialophora, Galerina, Psilocybe, Coprinellus	Ascomycota, Basidiomycota	
P-4	#108	10	6	1.67	3	2	0	0	0	10	Rhizophagus, Diversispora, Trichomonascus	Mucoromycota, Ascomycota	Apidaecin
P-5	#245	5	5	1.00	3	2	0	0	0	5	Pterula, Puccinia, Batrachochytrium	Basidiomycota, Chytridiomycota	FKBP_C
P-6	#205	5	4	1.25	2	2	0	5	0	5	Yarrowia, Cutaneotrichosporon	Ascomycota, Basidiomycota	CTD
P-7	#26	35	33	1.06	2	2	0	0	0	35	Talaromyces, Cryptococcus	Ascomycota, Basidiomycota	

^a^Corresponding to those in Fig. 5

^b^Corresponding to those in Additional file 3: Table S2

^c^Those having MF_alpha and/or MF_alpha_N HMM profiles as listed in Additional file 3: Table S2

^d^Those annotated as pheromones in the NCBI database but not “yeast a-mating factors”, as listed in Additional file 3: Table S2

^e^Those having the genes encoding DUF3328 domain-containing proteins within 15 genes

^f^Those having no genes encoding DUF3328 domain-containing proteins within 15 genes

^g^Omitted types including KEPs from 2 unclassified strains from this comparison

If some types of KEPs play vital roles in fungi, they should be distributed ubiquitously among different taxa. In Fig. 4b and c, the numbers of genera and phyla, respectively, to which the types of KEPs belong are shown in the same order as in Fig. 4a. A distinctive peak at around the 200th type (Fig. 4b) corresponds to 1.1 KEPs per type and strain, which suggests that the types playing indispensable and general roles in fungi are almost always encoded by a single-copy gene, with occasional duplications. The top 10 and 7 types ordered by the number of genera and phyla, respectively, are listed in Table 2, and the most abundant HMM profiles of their repeated sequences are shown in Fig. 5b and c, respectively. Type G-1/P-2 was the most ubiquitously distributed and was found in 49 genera and 2 phyla, followed by type G-2 (35 genera from Ascomycota). The top 2 types ordered by the number of phyla, G-5/P-1 and G-1/P-2, were also included in the top 10 types ordered by the number of genera, meaning that these types are ubiquitous across not only genera but also phyla. Probably reflecting this ubiquitous distribution, the G-1/P-2 and G-5/P-1 types were annotated with 26 and 11 Pfam motifs, respectively.

In the HMM profiles of the top 10 and 7 types by the number of genera and phyla, respectively, a typical feature was the presence of two to three W’s located close to each other with one other amino acid in between, as seen in G-4 and P-6 (Fig. 5b, c). The G-4 type comprised the highest number of KEPs (191), among which 174 were annotated as yeast α-mating factors because of the presence of the MF_alpha and MF_alpha_N Pfam domains; the HMM profile of the MF_alpha domain includes a WXW motif. Similarly, 121 KEPs of the G-2 type were annotated as fungal pheromones, which are homologous to the α-pheromone precursor peptides whose genes were experimentally validated to be expressed in five filamentous fungi (Table 2). Another distinctive feature was a di-histidine (HH) motif in G-3 and G-8. High content of Y was observed in S-3, S-5, G-1/P-2, G-5/P-1, G-6, G-10, and P-4, often accompanied by D in the vicinity. Multiple D’s in the same profile were observed in S-2, S-3, S-6, G-5/P-1, G-6, and P-7.

The common characteristics described above were found in the types distributed in several genera and phyla; however, the repeated sequences in the other types of KEPs were highly diverse. As shown on the outer ring in Fig. 3, some of 69 types with 15 or more KEPs were distributed across genera (shown in red, yellow, green, or blue), whereas most KEPs belonging to types with 14 or fewer members (dark grey) or stand-alone types (light grey) localized in a single genus. The distribution of types was also affected by the biased distribution of strains with their genomes sequenced, but nevertheless most types were found only in a single genus (Fig. 4b).

As exemplified by the precursor peptide of asperipin-2a (Fig. 1b), KEPs often have more than one type of repeated sequences per KEP. In the classified repeated sequences in each of the 838 types, 6278 different sub-types were found. After excluding sub-types containing 1 or 2 repeated sequences, 2513 sub-types remained. This high diversity of repeated sequences suggests a wide variety of linear or cyclic peptidyl compounds produced by fungi; more interestingly, fungi might produce different compounds cleaved out from a KEP depending on the stimulus or environmental situation, as observed for a mammalian hormone precursor peptide, pro-opiomelanocortin, which is processed in tissue-specific manners [35].

### Genes for DUF3328-domain-containing proteins are enriched around KEP-encoding genes

DUF3328-domain-containing proteins are indispensable for cyclization of the core peptides of ustiloxins [7, 18], asperipin-2a [13, 19], and phomopsins [16]. A computational survey of 20 *Aspergillus* strains detected more than 40 types of KEPs whose genes are accompanied by genes encoding DUF3328-domain-containing proteins within 10 kb [13]. To further examine the relationship between KEPs and DUF3328-domain-containing proteins in the Fungi kingdom, the functions of proteins encoded by the 15 genes closest to the KEP-encoding genes were surveyed.

**Fig. 6:**
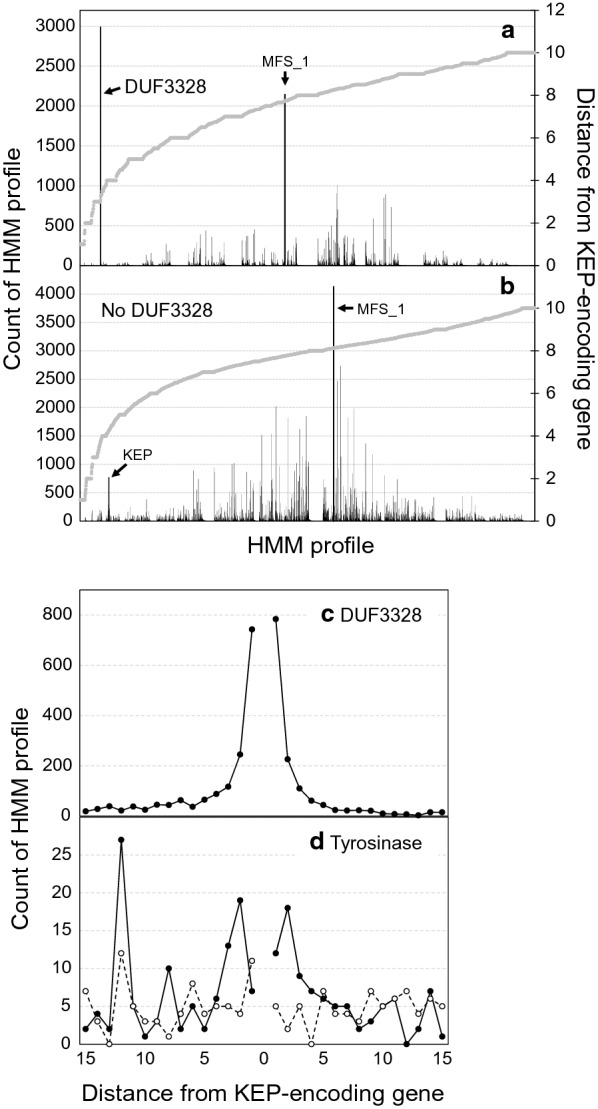
Counts of HMM profiles in the vicinity of genes encoding Kex2-processed repeat proteins (KEPs). The counts are shown as black bars separately for the cases when **a** genes for DUF3328-domain-containing proteins are present among the 15 genes closest to the KEP-encoding gene or **b** no such genes are present. The average distances of each HMM profile (up to 10 genes from a KEP-encoding gene) are shown as grey lines. The HMM profiles are ordered by the average distance from KEP-encoding genes, so the order differs in **a** and **b**. The counts of DUF3328 (**c**) and Tyrosinase (**d**) profiles are shown as solid lines with filled circles when genes for DUF3328-domain-containing proteins are present among the 15 genes closest to the KEP-encoding gene or dashed lines with open circles when otherwise, on the distance from KEP-encoding genes

Among the 7878 KEPs, the genes for 1758 (22%) were accompanied by genes for DUF3328-domain-containing proteins, located at an average distance of 3.09 genes (Fig. 6a). A total of 2991 genes for DUF3328-domain-containing proteins were detected in the vicinity of KEP-encoding genes (on average 1.7 DUF3382-domain-containing protein genes per KEP-encoding gene). This is in accordance with the presence of 2 and 5 DUF3328-domain-containing proteins in the biosynthetic pathways of ustiloxins [7] and phomopsins [16], respectively, whilst just one is present in that of asperipin-2a [13, 19]. The count of DUF3328-domain-containing protein genes sharply increased as approaching to KEP-encoding genes, and became maximum at the next position to the KEP-encoding gene (Fig. 6c). The accumulation of the cyclization factor genes around KEP-encoding genes is understandable by comparing it to that of the genes for MFS_1-containing proteins (major facilitator superfamily transporter), the most abundant HMM profile among the surveyed 1461 strains. The total count of MFS_1-containing protein genes within 15 genes from KEP-encoding genes was 2145 (0.7% of a total of 321,000 counts among the 1461 strains), and the average position was 7.74 genes from the KEP-encoding gene, whereas the count of DUF3328 was 31.8% of a total of 9399 counts and the average position was less than half of that of MFS_1-containing protein genes.

Ustiloxins, asperipin-2a and phomopsins are cyclized through ether bond formation directly in the aromatic ring of Y [7, 13, 16]. In accordance with this, 65.6% repeated sequences contained one or more Y’s in 1758 KEPs whose genes were accompanied by genes for DUF3328-domain-containing proteins. On the other hand, 38.2% repeated sequences contained one or more Y’s in 6120 KEPs whose genes were not accompanied by DUF3328-domain-containing protein genes. Among the abundant 23 types shown in Table 2 and Fig. 5, the KEP-encoding genes of seven types are accompanied by genes for DUF3328-domain-containing proteins (Fig. 5, labels in bold). Four of these seven types (S-5, G-5/P-1, G-6, and G-10) contain Y, whereas the other three (G-3, G-8, and P-3) contain di-histidine motifs (G-3 and G-8) or W (P-3) accompanied by P. Compared to the Y content rate, the HH and W content rates were similar between the KEPs whose genes were and were not accompanied by DUF3328-domain-containing protein genes; the HH content rates were 5.7% and 3.9%, and the W content rates were 20.5% and 24.5% in the 1758 and 6120 KEPs whose genes were and were not accompanied by the cyclization factor genes, respectively.

A tyrosinase is also required for cyclization of core peptides of ustiloxins and phomopsins [16, 18]. Compared to genes for DUF3328-domain-containing proteins, genes for proteins containing a Tyrosinase (PF00264) domain did not significantly accumulate around KEP-encoding genes (Fig. 6d). The total count of Tyrosinase-domain-containing protein genes among the 15 closest genes from the KEP-encoding gene was 341; this is 4.3% of 7878 KEPs. However, when DUF3328-domain-containing protein genes were within the 15 genes adjacent to the KEP-encoding gene, Tyrosinase-domain-containing protein genes also accumulated with ≈ 20 counts at the position of 2 genes from the KEP-encoding gene; such peaks of counts were not seen when genes for the cyclization factor were not among the 15 closest genes from the KEP-encoding gene (Fig. 6d). Accordingly, the average position of the Tyrosine-domain-containing protein genes was 6.46 genes from KEP-encoding genes accompanied by DUF3328-domain-containing protein genes, whereas that was 8.07 genes from KEP-encoding genes not accompanied by the cyclization factors. In the biosynthetic gene clusters of ustiloxins and phomopsins, the tyrosinase-encoding gene is respectively 7- and 8-gene distant from the precursor-peptide-encoding gene [7, 16]. Considering this, there can be other RiPP biosynthetic gene clusters that contain both a tyrosinase and DUF3328-domain-containing protein(s) for cyclization of RiPP core peptides.

Among the 6120 KEPs whose genes were not accompanied by genes for DUF3328-domain-containing proteins, 767 (9.7% of the total 7878 KEPs) were accompanied by another KEP-encoding genes at an average distance of 4.26 genes (Fig. 6b). The types of KEPs whose genes accompany each other have not been investigated; such investigation would be of interest. In this case, the total count of MFS_1 was 4132 (1.3%) at an average position of 8.13 genes from KEP-encoding genes.

As shown by red bars on the inner circle in Fig. 3, the KEP-encoding genes accompanied by genes for DUF3328-domain-containing proteins were detected mainly in Ascomycota. The number of such KEPs was 1647 out of 5372 (30.1%) in Ascomycota and 103 out of 1680 (6.1%) in Basidiomycota (Table 1). As the average number of KEPs per strain was approximately 5.3 in both Ascomycota and Basidiomycota, the cyclization factor motif sequence might differ between the two phyla, or cyclization of KEPs might hardly occur in Basidiomycota. In accordance with this, the content rate of Y in repeated sequences was 45.8% in Ascomycota, which is 1.3-times higher than that of 36.3% in Basidiomycota. Another remarkable observation was the absence of any DUF3328-domain-containing proteins in the Saccharomycetes class (Ascomycota). Thus, KEPs in Saccharomycetes, whose average number per strain was 1.35 and most of which were annotated as yeast α-mating factor, are processed and secreted probably in a linear form. In accordance with this, yeast α-mating factor is a linear peptide [26]. No DUF3328-domain-containing proteins were detected in any of the other six phyla.

### Mating factor genes are not accompanied by genes for DUF3382-domain-containing proteins

**Fig. 7:**
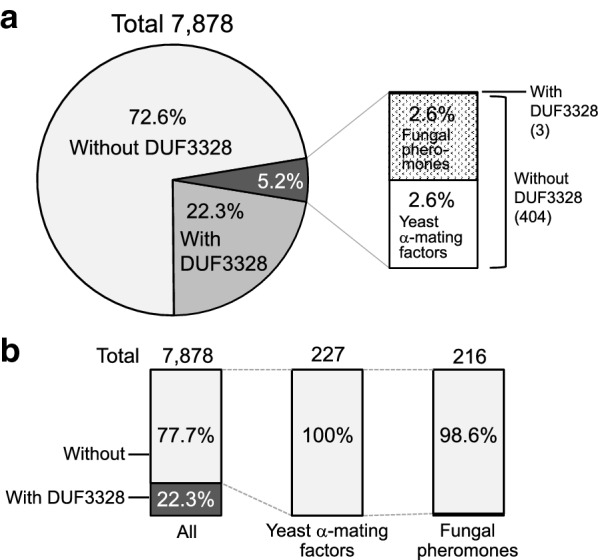
Breakdown of Kex2-processed repeat proteins (KEPs) according to the function and the presence of DUF3328-domain-containing protein genes in the vicinity. **a** A total of 7878 KEPs were broken down into those with (22%) or without DUF3328-domain-containing proteins and not annotated as yeast α-mating factors or other fungal pheromones (74%), and those annotated as yeast α-mating factors (3%) or other fungal pheromones (1%) are shown in the inset. Note that the inset breakdown includes one KEP whose gene is accompanied by a DUF3328-domain-containing protein gene. **b** All KEPs (left), those in the types containing the KEPs annotated as yeast α-mating factors (middle) or as other fungal pheromones (right) were broken down into those with or without DUF3328-domain-containing proteins

The breakdown of KEPs in the Fungi kingdom based on the results described above is shown in Fig. 7 in terms of the function as mating factors and the presence of genes for DUF3328-domain-containing proteins in the vicinity. Among a total of 7878 KEPs, the genes of 22.3% were accompanied by DUF3328-domain-containing protein genes. Among all KEPs, 2.6% (203) are annotated as yeast α-mating factors and another 2.6% (204) as fungal pheromones (Fig. 7a, Additional file 3: Table S2), in accordance with the previous study [25]. None of the genes for KEPs annotated as yeast α-mating factors were accompanied by genes for DUF3328-domain-containing proteins. The situation is the same for KEPs annotated as fungal pheromones with three exceptions as described below. In addition to the most abundant KEP type, G-4, in which 174 of the 191 KEPs were annotated as yeast α-mating factors, KEPs of two other types were also annotated to have Pfam MF_alpha and/or MF_alpha_N domains, corresponding to yeast α-mating factor and its N-terminal region, respectively. The 204 putative fungal pheromones come from 5 types. Overall, 227 and 216 KEPs are annotated as groups of yeast α-mating factors and fungal pheromones, respectively. As mentioned above, genes for 22.3% of all 7878 KEPs were accompanied by DUF3328-domain-containing protein genes; genes for 100% of the putative 227 yeast α-mating factors and 98.6% of the 216 putative fungal pheromones were not accompanied by a cyclization factor gene (Fig. 7b). The three putative fungal pheromones whose genes were accompanied by cyclization factor genes were GAQ44527.1 from *Aspergillus niger*, PWY62495.1 from *Aspergillus eucalypticola*, and PQE12560.1 from *Rutstroemia* sp.; the genes for DUF3328-domain-containing proteins were located at a distance of 15, 12, and 2 genes, respectively, from the KEP-encoding genes. As the average distance of DUF3328-domain-containing protein genes from KEP-encoding genes was 3.09, the former two cyclization factors might not function in the modification of the KEPs and the location of their genes in the vicinity may be coincidental.

The group of putative fungal pheromones containing 216 KEPs came only from ascomycetes (214) and basidiomycetes (2). One of the main reasons for this restricted distribution is probably that KEPs were assigned to fungal pheromones by the homology with five Ascomycota α-pheromone precursor peptides, whose genes were experimentally validated to be expressed (EAL88490.1, *A. fumigatus* PpgA [36]; CAB96172.1 from *S. macrospora* Ppg1 [29, 37]; AAC64288.2, *N. crassa* Ccg-4 [28]; EHA53132.1, *Magnaporthe grisea* MF2-1 [38]; AAC39328.1, *Cryphonectria parasitica* Mf1/1 [39]). Marquer et al. detected some KEPs possessing a repetition of STE13-cleaved dipeptide in Basidiomycota, indicating that those KEPs might function as unknown mating factors in Basidiomycota [25]. In this study, 76 KEPs of 7878 were homologous to the six putative Basidiomycota pheromones shown in the previous study [25], but the genes for 22% (17) of them were accompanied by genes for DUF3328-domain-containing proteins unlike KEPs of putative yeast α-mating factors and putative Ascomycota α-pheromone precursors.

## Discussion

The computational genome mining revealed that KEPs are widely distributed in the Fungi kingdom, but their sequence types are highly diverse and most of them are unique at least at the genus level. The 7878 KEPs detected in 1345 out of 1461 strains were classified into 838 types and 2560 stand-alone KEPs, and most types were further classified into a total of 6278 sub-types. Genes for 22% of the KEPs were accompanied by genes for cyclization factors (DUF3328-domain-containing proteins); however, the genes for 227 factors annotated as yeast α-mating factors and for 216 factors annotated as fungal pheromones were not, with three exceptions. The Y content rate was 65.6% in repeated sequences of KEPs whose genes were accompanied by genes for cyclization factors, whereas that was 38.2% when the KEP-encoding genes were not accompanied by genes for cyclization factors. The results indicate that fungi produce a wide variety of cyclic and linear peptidyl compounds, which are likely to have important functions in fungal ecology, as suggested by their wide conservation in the Fungi kingdom. As fungi, animals are eukaryotes and they secrete many peptides as neuropeptides (hormones) cleaved out from precursors (prohormones). For example, thyrotropin-releasing hormone [40], as well as opiomelanocortin [35], is cleaved out from its prohormone into several different hormones by different processing enzymes depending on tissues, and the cleavage sites are KR, KK and RK. Oxytocin and Gonadotropin are cleaved out from its precursor at GKR [41, 42].

**Fig. 8:**
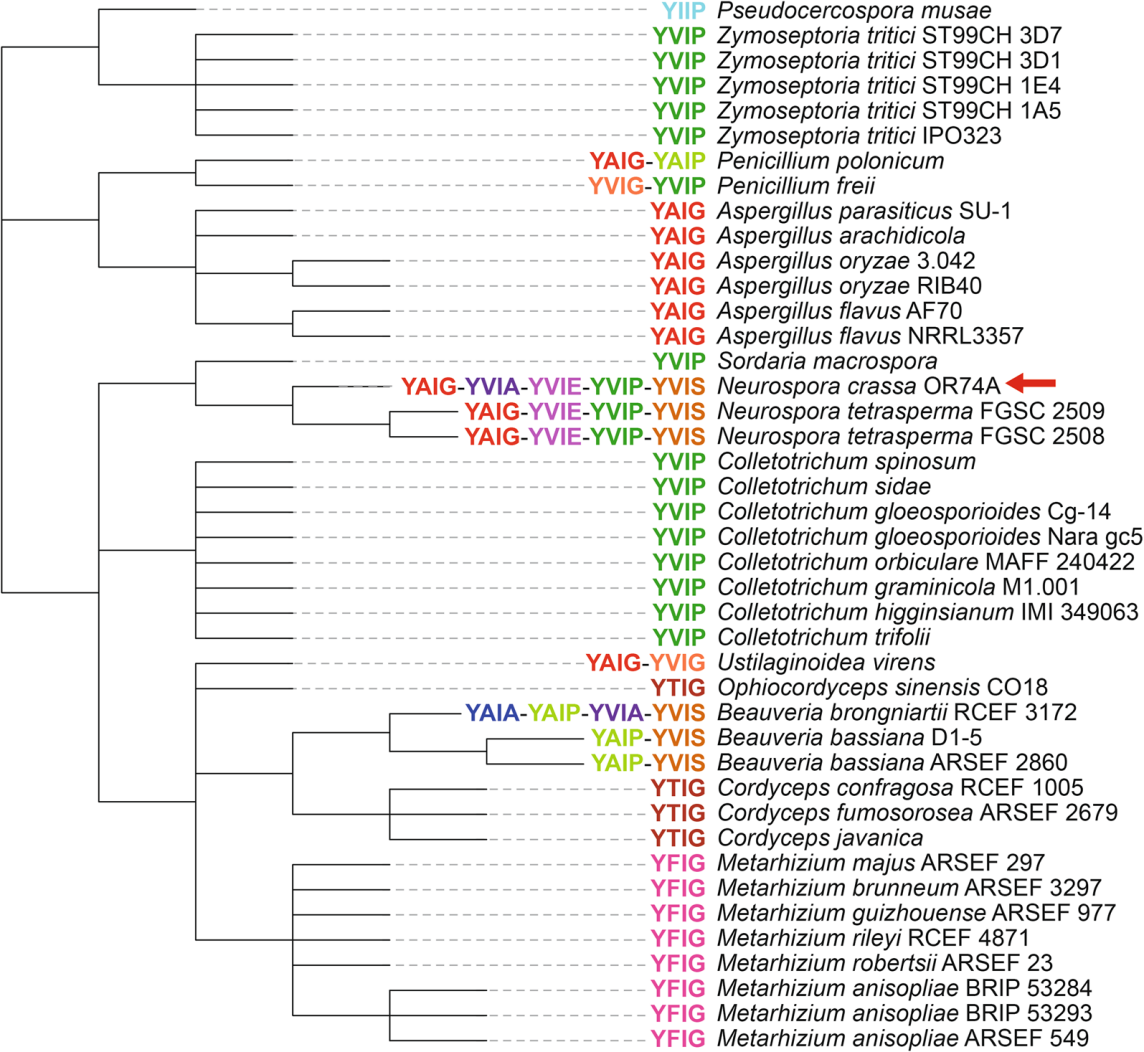
Taxonomic tree of strains containing Kex2-processed repeat proteins (KEPs) homologous to the ustiloxin precursor peptide. Core peptide sequences deduced from those of ustiloxins and phomopsins are shown next to the strains. Red arrow indicates the POI2 protein, which is experimentally shown to be involved in sexual structure formation in *Neurospora crassa*

**Fig. 9:**
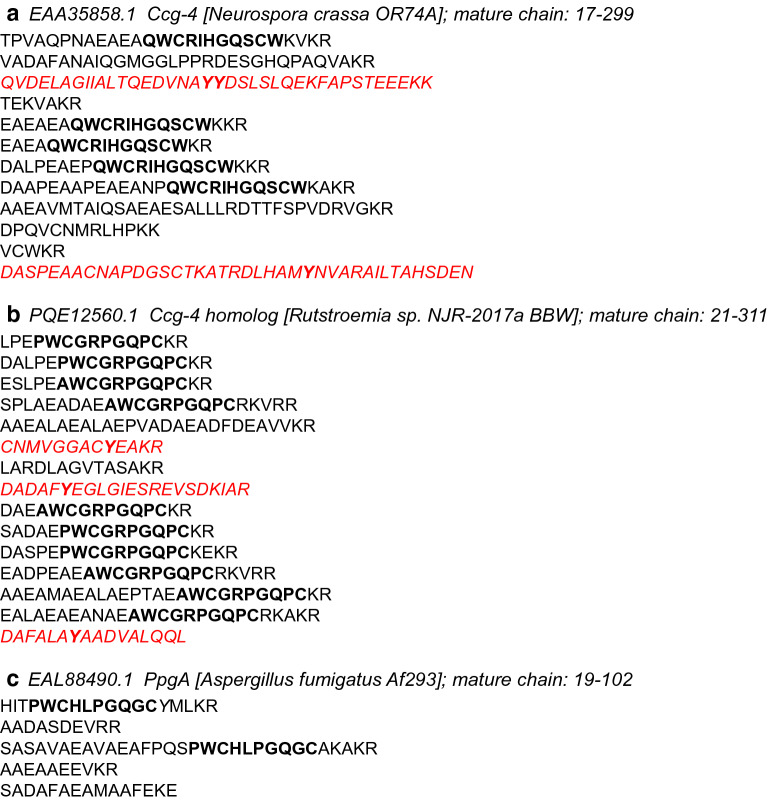
Representative sequences of Kex2-processed repeat proteins (KEPs) that function as fungal pheromones. Signal peptides were trimmed. **a** Ccg-4 of *N. crassa*, which is experimentally shown to be regulated by the mating type locus; its gene is not accompanied by DUF3328-domain-containing protein genes. **b** Ccg-4 homolog of *Rutstroemia*; its gene is accompanied by a DUF3328-domain containing protein gene. **c** PpgA of *Aspergillus fumigatus*; its gene is not accompanied by DUF3328-domain containing protein genes. The presumed pheromone peptides are shown in bold. The fragmented sequences cleaved by Kex2 containing no pheromone peptides but Y (in bold) are shown in red

**Fig. 10:**
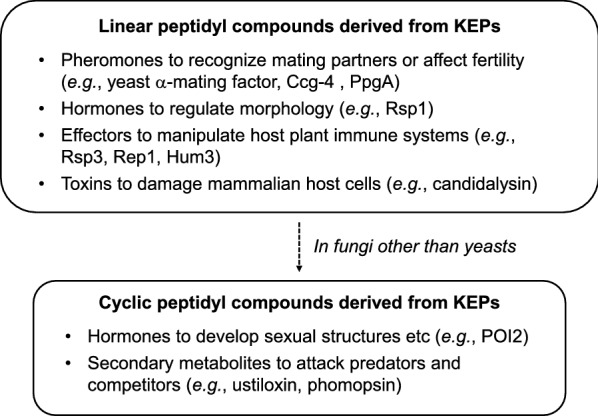
The summary and hypothetical evolutionary pathway of Kex2-processed repeat proteins (KEPs)

The yeast α-mating factor is the first identified peptide from a KEP [43], and some other linear peptides from KEPs have been experimentally verified as fungal effectors to manipulate host plants [20, 22, 23], the factor for aerial hyphae formation [21] and a cytolytic toxin to damage mammalian epithelial cells [24]. Compared to linear peptides from KEPs, an understanding of the function of cyclic compounds from KEPs in fungal ecology is lacking. One clue to the function of cyclic peptides synthesized from KEPs was reported for *N. crassa* POI2 [44], which is a KEP homologous to the precursor peptides of ustiloxin and phomopsin. Kim and Nelson provided experimental evidence that POI2 is essential for differentiation of female reproductive structures and perithecial development, as well as for normal vegetative growth [44]. Figure 8 shows a taxonomic tree of the strains possessing the same type of KEPs as POI2. In the sequence motif of this type (Fig. 5b, G-10), Y and I are conserved (with three exceptions in a total of 441 repeated sequences, where all three amino acids after Y were deleted), as well as in the core peptides YAIG (ustiloxin B), YVIG (ustiloxin A [8]) and YVIP (phomopsin), in accordance with cyclization of the core peptides of ustiloxin and phomopsin at these Y and I. Based on this motif, the tetra-peptide YXIX in the repeated sequences appears to be the core peptide, which is converted into the compounds derived from KEPs (Fig. 8). The *Aspergillus*, *Colletotrichum*, *Cordyceps*, and *Metarhizium* genera each have a single core peptide conserved among species. On the other hand, *Penicillium*, *Neurospora*, *Ustilaginoidea*, and *Beauveria* each have more than one kind of core peptide, and their combinations vary among species within the genus, except in *Ustilaginoidea*, which is represented by only one strain. In these genera, more than one type of cyclic compound might be necessary to regulate sexual development, and the combinations of the core peptides may serve as the signatures of the species. The core peptides in *Zymoseptoria*, *Sordaria*, and *Colletotrichum* are all YVIP, even though these genera are not taxonomically close to each other. This core peptide is also the same as that of phomopsins, which is toxic to mammalian cells because it acts as a tubulin inhibitor [16], which is also a property of ustiloxin (YAIG) [30, 31]. The toxicity of the compounds derived from the core peptides YTIG and YFIG is unknown, but if they are also toxic, these compounds, derived from KEPs containing one type of core peptide, might have evolved to act as secondary metabolites (*e.g.*, biosynthesized by co-regulated genes in a cluster) owing to their strong bioactivities.

Another clue to the possible evolutionary pathway of KEPs is provided by the pheromone precursor Ccg-4 in *N. crassa* OR74A (EAA35858.1) and its homolog in *Rutstroemia* sp. NJR-2017a BBW (PQE12560.1) (Fig. 9). *Neurospora crassa* Ccg-4 is regulated by the mating-type locus [28] and is essential for male fertility [45]. There are no genes for DUF3328-domain-containing proteins among 15 genes closest to *N. crassa ccg*-*4*, whereas the *Rutstroemia* homolog has one at the position of 2 genes. The *N. crassa* and *Rutstroemia* proteins have 12 and 15 sequences, respectively, separated by Kex2 recognition sites, among which pheromone-like sequences and other sequences are mixed. Y is mandatory for cyclization by DUF3328-domain-containing proteins in ustiloxins, phomopsin and asperipin-2a [16, 18, 19]; *N. crassa* Ccg-4 has two fragmented sequences (38 and 40 a.a.) containing Y, whereas the *Rutstroemia* Ccg-4 homolog has three (13, 16, and 22 a.a.). The lengths of the repeated sequences are typically 13, 14, and 8 a.a. in the precursor peptides of ustiloxin, phomopsin, and asperipin-2a, respectively (Fig. 1). Considering this, the *Rutstroemia* Ccg-4 homolog might have initially contained a core peptide only for a fungal pheromone, and might have gradually evolved to produce a cyclic compound under the regulation of the mating-type locus. The *A. fumigatus* fungal pheromone, PpgA (EAL88490.1), has no repeated sequences containing Y other than pheromone-like sequences (Fig. 9).

Based on these observations and speculations, a hypothetical evolutionary pathway of KEPs in the Fungi kingdom is suggested (Fig. 10). KEPs are a group of secretory proteins as a fungal armory to survive in ecosystem. Some linear peptidyl compounds derived from KEPs began to be used as fungal pheromones to recognize partners to start mating or to affect fertility under the regulation of the mating type locus, whereas some others to infect host cells as effectors or toxins. The pheromones might be used to distinguish mating partners of the same genus (*e.g.*, to distinguish *Neurospora* and *Aspergillus*), whereas various homologs within each type of KEPs might be used to identify the correct partner of the same species among hundreds of species in a genus (*e.g.*, distinguishing *N. crassa* and *N. tetrasperma*). Except in Saccharomycetes (yeasts) and Tremellomycetes (jelly fungi), the genes for some KEPs became accompanied by genes for DUF3328-domain-containing proteins, and some cyclic peptidyl compounds started to be produced for cell differentiation induced by mating, whilst some other cyclic compounds that had strong bioactivities (*e.g.*, toxicities) began to be regulated as secondary metabolites against competitive microorganisms and predatory organisms like animals and nematodes. For example, amanitins, the first reported RiPPs in fungi produced by *Amanita* mushroom [9], have a strong toxicity against animals as an RNA polymerase II inhibitor [46]. It is unclear that either of pheromone-type or effector-type linear peptides from KEPs evolved first or each type was evolved independently, but most yeasts have only KEP as a mating factor. Similarly, it is unknown if either hormone-type or toxin-type of cyclic compounds from KEPs evolved first or each type did independently, but some studies indicated a relation between sexual regulation and secondary metabolism [47, 48]. Analyzing sequence evolution of KEPs along with the expression patterns of genes around KEP-encoding genes will help to shed light on these points.

## Conclusion

KEPs are widely distributed in the Fungi kingdom, but their repeated sequences are highly diverse. A total of 7878 KEPs were distributed in 1345 out of 1461 strains belonging to 8 phyla. The average number of KEPs per strain was 5.25 in ascomycetes and 5.30 in basidiomycetes, but was distinctively small in the classes Saccharomycetes (1.35) and Tremellomycetes (1.00). The KEPs were classified into 838 different types and 2560 stand-alone KEPs, which have no homologs. Nearly 200 types were found in more than one genus, and 14 types in more than one phylum. The types distributed among genera and phyla included yeast α-mating factors and fungal pheromons. The genes for 22% of all KEPs were accompanied by genes for DUF3328-domain-containing proteins, which are indispensable for cyclization of core peptides to produce ustiloxin-type compounds, within an average distance of 3.09 genes; however, except in 3 cases, all KEPs annotated as yeast α-mating factors or fungal pheromones had no genes for DUF3328-domain-containing proteins in the vicinity. From these results and some examples, the evolutionary pathway of KEPs was hypothesized that first they might have evolved in an unmodified linear form (*e.g.*, mating factors), and then those in a modified cyclic form emerged (*e.g.*, toxins).

## Methods

### Genome assemblies

The latest whole-genome assemblies of 1461 strains belonging to the Fungi kingdom were downloaded from the National Center for Biotechnology Information (NCBI) database on October 9, 2019. The assembly accessions are listed in Additional file 1: Table S1, together with information on the strain, genus, phylum, taxonomy ID, and the number of KEPs detected.

### Detection of KEPs

Among the 14,491,621 protein sequences annotated in the 1461 assemblies, those having signal peptides for translocation into the ER were identified using the SignalP-4.1 algorithm [49]. The resulting 1,123,012 protein sequences were narrowed down to 69,264 by detecting tandem-repeat sequences. Briefly, a seed sequence of 40 a.a. in length was cut out from a protein sequence, from which the signal peptide had been removed based on the results of SignalP, and aligned to the original sequence by shifting the initial position one by one. Each amino acid in the original protein sequence was scored when it was identical to the amino acid in the seed sequence. The position of the seed sequence on the original sequence was shifted for as many steps as possible. The score of each amino acid was divided by the number of the seed sequence used, and the amino acid positions were regarded as repeated when the score was greater than 0.6. If the original protein sequence was divided into more than 2 parts at the repeated positions, the protein was considered as having tandem repeats. Among 69,264 such proteins, 7878 were regarded as KEPs because they had ≥ 3 tandem repeats of ≥ 8 a.a. each between the Kex2 recognition sites (KR, KK, RK, or RR), but any repeated sequence(s) were not longer than 100 a.a. The detected KEPs are listed in Additional file 3: Table S2 with the information on their strain, genus, phylum, FRiPS type, indices, and HMM profiles (the latter three were evaluated as described below). All calculations were performed in Ubuntu version 18.04.3 using in-house codes written in Perl or Python languages in this study, unless specific algorithms are mentioned.

The detected KEPs were thoroughly compared with KEPs detected in the previous study [25] as follows. First, the strains surveyed in the previous study were considered as identical to those surveyed in this study when their species taxonomy ID’s were the same. Next, the similarity between the KEPs in the overlapped strains were evaluated using the SSEARCH program [50], which implemented the Smith–Waterman algorithm [51], and KEPs in this study were considered as identical to those in the previous study [25] when their identity and coverage were more than 95%.

### Classification of KEPs

The 7878 KEPs were classified into 838 types according to the e-values returned by a BlastP search [52] against a database containing all these KEPs. Two KEPs were paired as the same type when they had e-values less than 1E−30 and the coverage of the high-scoring pair over the original sequence was more than 70% in reciprocal BlastP searches. The pairs were combined into one type when they shared at least one KEP, and each type was labeled by the order of the number of KEPs belonging to the type (Additional file 3: Table S2, “KEP type” column, and Table 2, “Type ID” column). The stand-alone KEPs having no homologs were assigned to type “0”.

KEPs frequently have more than one type of repeated sequences in the same protein. To group homologous repeated sequences in every type of KEP, the similarity between every two repeated sequences in every type of KEP was evaluated using the SSEARCH program [50]. Two repeated sequences were paired when their identity and coverage in reciprocal alignments were more than 65% and 70%, respectively, and were classified into the same group in the same manner as KEPs were classified in the same type. The repeated sequences belonging to the same group in the same type of KEPs were aligned using the MAFFT program (version 7.299) with the “–auto” option [53], and then HMM profiles were generated using the hmmbuild module in HMMer version 3.3 [54] with the “–amino” option. For some repeated sequences in the most common types of KEPs, logos representing both the sequence alignment and the HMM profile were generated from the HMM profiles on the Skylign website (https://skylign.org/) [55].

### Generation of taxonomic tree

The taxonomic tree of the 1461 strains examined in this study was generated at the genus level on the NCBI Taxonomy Common Tree website (https://www.ncbi.nlm.nih.gov/Taxonomy/CommonTree/wwwcmt.cgi) using the list of the strains’ taxonomy IDs and the taxonomic relationship described in the “nodes.dmp” file stored in the NCBI taxonomy database. The tree was drawn on the iTOL web server (https://itol.embl.de/) [56]. Information on the phyla and average numbers of KEPs per genus was depicted using colored circle segments and bars outside the tree, respectively. The bars were colored by the type of KEPs.

### Functional analyses of KEPs and proteins encoded by nearby genes

The functional motifs in KEPs and proteins encoded by 15 genes closest to the KEP-encoding gene were searched by using the hmmscan module in HMMer version 3.3 with a threshold of 1E−5 against the Pfam database release 32.0 [57]. The HMM profiles detected were counted, and the total counts and average distances from KEP-encoding genes were evaluated separately for the cases when KEP-encoding genes are accompanied or not by genes for DUF3328-domain-containing proteins. KEPs whose genes were accompanied by genes for DUF-3328-domain-containing proteins within 15 genes were marked with “x” in Additional file 3: Table S2, “DUF3328 in vicinity” column. The correlation between the existence of Y in KEP repetitive sequences and DUF3328-domain-containing proteins encoded by 15 genes closest to the KEP-encoding gene were observed by the percentage of KEP repetitive sequences in length from 8 a.a. to 100 a.a. containing one or more Y’s.

### Annotation of yeast α-mating factors, fungal pheromones and Basidiomycota pheromones

When a KEP had the Pfam annotations of MF_alpha (Pfam ID PF04648) and/or MF_alpha_N (PF05436), which are the motifs of yeast α-mating factor and its N terminus, respectively, this KEP was assigned to yeast α-mating factors. A KEP was annotated as fungal pheromones when it had an e-value less than 1E−10 by a BlastP search [52] using queries of five fungal α-pheromone precursor peptides (EAL88490.1, *A. fumigatus* PpgA [36]; CAB96172.1 from *S. macrospora* Ppg1 [29, 37]; AAC64288.2, *N. crassa* Ccg-4 [28]; EHA53132.1, *M. grisea* MF2-1 [38]; AAC39328.1, *C. parasitica* Mf1/1 [39]). Similarly, a KEP was annotated as Basidiomycota pheromones when it had an e-value less than 1E−10 by a BlastP search [52] using queries of six Basidiomycota α-pheromone precursor peptides shown in Fig. 7b (jgi|Exigl1|760266|gm1.2247_g, jgi|Fomme1|148261|estExt_fgenesh1_pg.C_90156, jgi|Lacbi2|334723|Lacbi1.fgenesh3_pg.C_scaffold_68000056, jgi|Mixos1|81973|fgenesh1_pg.4_#_177, jgi|PleosPC15_2|1091723|estExt_fgenesh1_pm.C_011030, and jgi|Triol1|112374|CE112373_296. These three annotations of yeast α-mating factors, fungal pheromones and Basidiomycota pheromones were marked with “x” for KEPs in Additional file 3: Table S2, “Yeast α-mating factor”, “Fungal pheromones” and “Basidiomycota pheromones” columns, respectively.

### Analyses of ustiloxin precursor peptide homologs among KEPs

In addition to 45 KEPs belonging to the same type as the *A. flavus* ustiloxin precursor peptide (EED49417.1), 10 KEPs belonging to the same types as putative dikaritin biosynthetic precursor peptides in *Colletotrichum* [16] (CCF41414.1, EFQ36603.1, ELA31405.1, and EQB43629.1) were considered as ustiloxin-type KEPs. The taxonomic tree was generated for the strains containing these 55 ustiloxin-type KEPs using the NCBI taxonomy Common Tree website, and drawn on the iTOL web server.

## Supplementary information

**Additional file 1: Table S1.** The genome assemblies used in this study with the number of KEPs detected.

**Additional file 2: Figure S1.** The KEPs detected in this study.

**Additional file 3: Table S2.** The KEPs detected in this study.

**Additional file 4: Table S3.** The 20 KEPs of type S-1 in Peniophora sp. CBMAI 1063 as the example suggesting gene duplication1.

## Data Availability

The lists of surveyed assemblies and detected KEPs are provided as Additional file 1: Tables S1 and Additional file 2: Table S2, respectively.

## Abbreviations

RiPP: Ribosomally synthesized and post-translationally modified peptide
KEP: Kex2-processed repeat protein
HMM: Hidden Markov model
